# Regulation of SLC7A11 as an unconventional checkpoint in tumorigenesis through ferroptosis

**DOI:** 10.1016/j.gendis.2024.101254

**Published:** 2024-03-02

**Authors:** Zhenyi Su, Yanqing Liu, Lin Wang, Wei Gu

**Affiliations:** a Institute for Cancer Genetics, Department of Pathology and Cell Biology, Herbert Irving Comprehensive Cancer Center, Vagelos College of Physicians & Surgeons, Columbia University Irving Medical Center, New York, NY 10032, USA; b Center for Translational & Computational Neuroimmunology, Department of Neurology, Columbia University Irving Medical Center, New York, NY 10032, USA

**Keywords:** Ferroptosis, Metabolic checkpoint, SLC7A11, Stress, Targeted cancer therapy, Transcriptional regulation

## Abstract

Although cell-cycle arrest, senescence, and apoptosis are well accepted as the classic barriers in tumorigenesis, recent studies indicate that metabolic regulation is equally important as a major checkpoint in cancer development. It is well accepted that ferroptosis, an iron-dependent programmed cell death, acts as a new type of tumor suppression mechanism tightly linked with numerous metabolic pathways. SLC7A11 is a transmembrane cystine/glutamate transporter protein that plays a vital role in controlling ferroptosis *in vivo*. The levels of SLC7A11 are dynamically regulated by various types of stresses, such as oxidative stress, nutrient deprivation, endoplasmic reticulum stress, radiation, oncogenic stress, DNA damage, and immune stress. SLC7A11 can be transcriptionally regulated by both activators such as ATF4, NRF2, and ETS1, and repressors including BACH1, p53, ATF3, and STAT1 during stress responses. Moreover, SLC7A11 activity and its protein stability and cellular localization are also modulated upon stress. Patients' data show that SLC7A11 is overexpressed in various types of human cancers, and higher levels of SLC7A11 predict poorer overall survival. Growing evidence also suggests that targeting SLC7A11 is a promising approach in cancer therapy by effectively inhibiting tumor proliferation, invasion, and metastasis, as well as counteracting cancer stem cells and overcoming chemoresistance. This review highlights the regulation of SLC7A11 as an unconventional checkpoint in tumorigenesis through modulating ferroptotic responses under various types of stress.

## Introduction

Ferroptosis is an iron-dependent regulated cell death driven by the accumulation of toxic lipid peroxides.^1^, ^2^, ^3^ Unlike other forms of cell death that rely on certain executor proteins such as caspases in apoptosis, mixed lineage kinase domain-like protein in necroptosis, and gasdermins in pyroptosis, ferroptosis has distinct molecular mechanisms.^4^, ^5^, ^6^ During ferroptosis, a critical event is the peroxidation of polyunsaturated fatty acids (PUFAs) within cell membranes. This process generates lipid peroxides that can spread and amplify the damage to neighboring molecules and cellular structures. As lipid peroxides accumulate, they progressively undermine the integrity and functionality of cell membranes, resulting in cellular malfunction and, ultimately, the demise of the cell.^7^ Increasing evidence suggests that ferroptosis plays an important role in both physiological and pathological processes including tumor surveillance, immune regulation, neurodegeneration, ischemic damage, liver diseases, and cardiovascular disease.^3^^,^^8^, ^9^, ^10^, ^11^

In the past few years, many studies reported that various stresses including oxidative stress, oncogenic stress, nutrient deprivation, DNA damage, radiation, and inflammatory microenvironment can regulate ferroptosis.^3^^,^^12^, ^13^, ^14^, ^15^, ^16^ SLC7A11, also known as xCT, is a subunit of the antiporter of cystine and glutamate on the plasma membrane and serves as a central negative regulator in ferroptosis.^17^^,^^18^ Of importance, SLC7A11 expression and activity exhibit a high sensitivity to a wide range of stressors, which positions it as a central node connecting ferroptosis with various types of stress. In this review, we present recent progress in understanding ferroptosis induction and defense systems and summarize the latest advancements in SLC7A11 research, including its structure, regulation, expression profiles, and prognosis in cancer patients. We particularly focus on how various stresses regulate SLC7A11 transcription, protein stability, activity, and cellular localization, as well as the connections between these regulations and ferroptosis. Finally, we systematically review recent developments in SLC7A11-targeted cancer therapy.

## Dynamics of ferroptosis

In normal cellular metabolism, PUFAs in cell membranes may undergo some degree of lipid peroxidation. However, this may not end up with ferroptosis, because there is a delicate balance between ferroptosis inducing and ferroptosis defense systems in cells. Due to the presence of the antioxidant systems, this lipid peroxidation is effectively controlled within acceptable limits and does not cause severe damage to cells. In some circumstances, the cellular defense mechanisms against lipid peroxidation are compromised, leading to an accumulation of peroxides and subsequent damage to cell membranes and organelles.^19^^,^^20^

### Ferroptosis inducing system

#### Polyunsaturated fatty acid-phospholipid (PUFA-PL) synthesis and peroxidation

PUFA-PL synthesis and peroxidation are the material basis for ferroptosis. PUFA-PL synthesis critically relies on acyl-coenzyme A (CoA) synthetase long-chain family member 4 (ACSL4) and lysophosphatidylcholine acyltransferase 3 (LPCAT3).^21^, ^22^, ^23^ ACSL4 plays a crucial role in catalyzing the binding of free PUFAs, including arachidonic acids and adrenic acids, to CoA molecules. This enzymatic reaction results in the formation of PUFA-CoAs, such as arachidonic or adrenic acid-CoA.^21^^,^^22^ After the generation of PUFA-CoAs, these compounds are further processed by LPCAT3. LPCAT3 plays a crucial role in the re-esterification process, wherein PUFA-CoAs are incorporated into phospholipids. This enzymatic activity leads to the formation of PUFA-PLs, such as arachidonic or adrenic acid phosphatidylethanolamine.^23^ In addition, acetyl-CoA carboxylase is also involved in the regulation of ferroptosis by catalyzing acetyl-CoA to malonyl-CoA which is necessary for the synthesis of some long PUFAs.^23^^,^^24^ Indeed, deactivation of ACSL4, LPCAT3, or acetyl-CoA carboxylase can completely or partially block ferroptosis.^21^^,^^22^^,^^24^

Due to the presence of bis-allylic moieties in PUFAs, PUFA-PLs are highly vulnerable to peroxidation.^7^ The peroxidation of PUFA-PLs can be divided into two categories: non-enzymatic reaction and enzymatic reaction. Non-enzymatic autoxidation induced by the iron-mediated Fenton reaction is thought to be the major driver for PUFA-PL peroxidation, while cytochrome P450 oxidoreductase-mediated or lipoxygenase-mediated enzymatic reactions have also been reported to promote lipid peroxidation.^7^^,^^11^^,^^25^, ^26^, ^27^, ^28^, ^29^

#### Iron metabolism

Iron is critically important in the regulation of ferroptosis. On the one hand, iron-initiated non-enzymatic Fenton reaction plays a crucial role in governing ferroptosis by enabling the direct peroxidation of PUFA-PLs.^7^^,^^30^ On the other hand, iron acts as an essential cofactor for enzymes involved in lipid peroxidation, such as lipoxygenases and cytochrome P450 oxidoreductase.^30^^,^^31^ Cells precisely control the uptake, utilization, storage, and export of iron to maintain a stable pool of labile/free iron. Disruption or interference of any of these processes may increase or decrease the labile iron levels in cells to trigger or extinguish ferroptosis. A few iron transporters or carrier proteins are particularly important in modulating ferroptosis. For example, transferrin is a glycoprotein that binds tightly to iron in the blood and transports it to different tissues and organs.^32^ Transferrin's iron-binding properties are influenced by changes in pH, and it plays a crucial role in inducing ferroptotic cell death under conditions of amino acid starvation.^16^ Transferrin receptor is a receptor protein on the cell surface that binds to iron-loaded transferrin and helps transport iron into cells. Increased expression of transferrin receptors in cancer cells is positively correlated with their response to ferroptosis-inducing agents, making it a potential biomarker for predicting ferroptosis sensitivity in cancer treatment strategies.^33^ Moreover, ferritin is a cytosolic protein found in cells that acts as a reservoir for iron, which protects cells from ferroptosis.^34^ Conversely, nuclear receptor coactivator 4 is a protein involved in ferritinophagy, a selective autophagic process responsible for degrading ferritin.^35^ It mediates the degradation of ferritin, leading to iron accumulation in cells and induction of ferroptosis. It plays a crucial role in regulating iron homeostasis and has implications for various diseases, including cancer and pulmonary diseases.^36^

#### Mitochondrial metabolism

Mitochondrial activities contribute to ferroptosis from at least three aspects: First, mitochondria are a predominant source of cellular reactive oxygen species. Specifically, electron leakage from complexes I and III of the electron transport chain leads to the production of superoxides that are subsequently converted to H_2_O_2_ by the enzyme superoxide dismutase.^37^ H_2_O_2_ can further react with labile iron through the Fenton reaction, resulting in the formation of hydroxyl radicals. These hydroxyl radicals are responsible for driving the peroxidation of PUFA-PLs.^7^

Second, ATP production in mitochondria also contributes to ferroptosis.^24^^,^^38^ Under ATP-depleted conditions, AMP-activated protein kinase phosphorylates and inactivates acetyl-CoA carboxylase, thereby suppressing PUFA-PL synthesis and blocking ferroptosis. Conversely, with sufficient energy and ATP, AMP-activated protein kinase cannot be activated efficiently and acetyl-CoA carboxylase is activated, thus promoting PUFA-PL synthesis and ferroptosis.^24^^,^^38^

**Figure 1:**
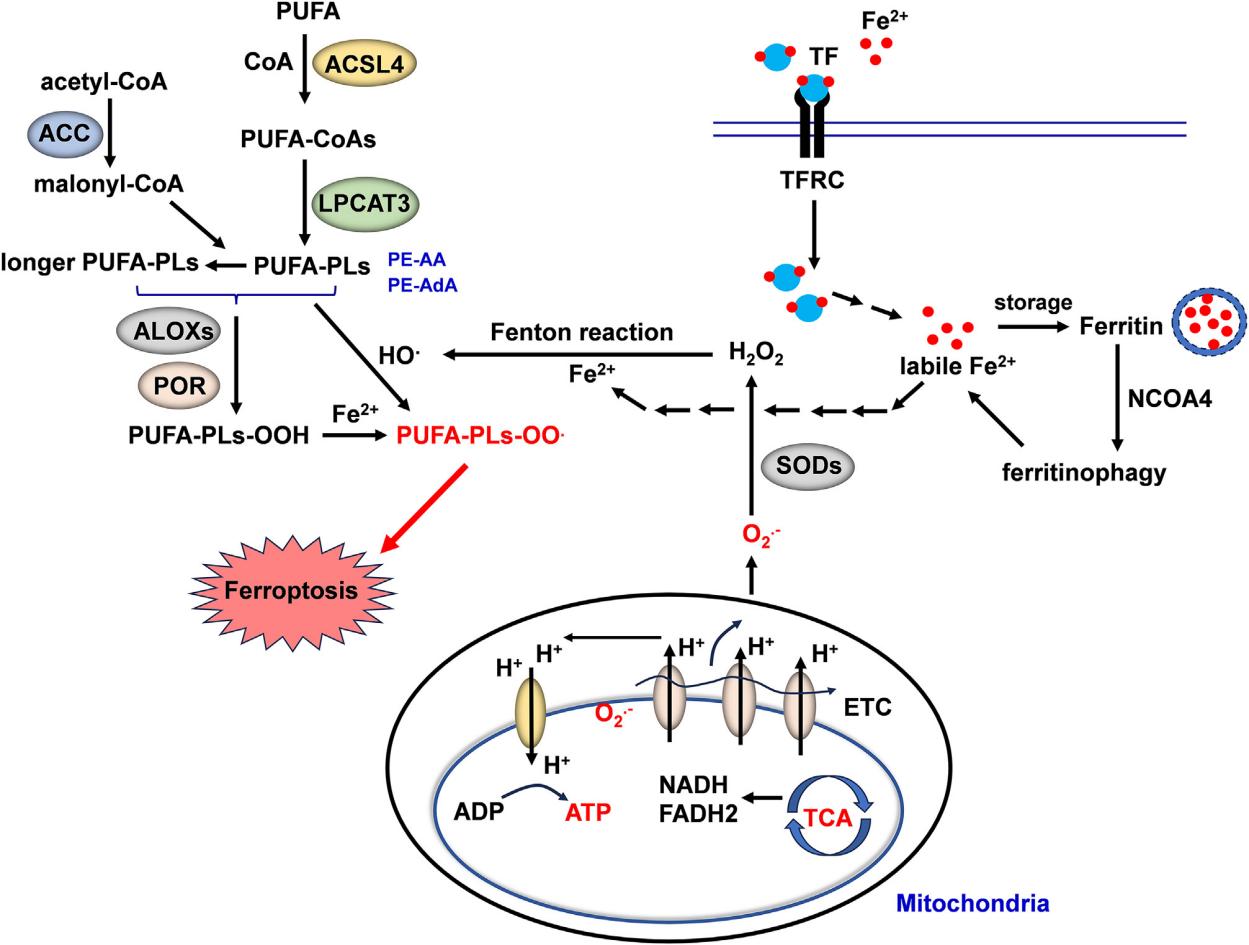
Ferroptosis inducing system. Polyunsaturated fatty acid-phospholipid synthesis and peroxidation, iron metabolism, and mitochondrial metabolism provide essential conditions for ferroptosis. ACC, acetyl-CoA carboxylase; ACSL4, acyl-coenzyme A (CoA) synthetase long-chain family member 4; ALOXs, lipoxygenases; ETC, electron transport chain; LPCAT3, lysophosphatidylcholine acyltransferase 3; NCOA4, nuclear receptor coactivator 4; O2^.−^, superoxide radical; PE-AA, phosphatidylethanolamine-arachidonic acid; PE-AdA, PE-adrenic acid; POR, cytochrome P450 oxidoreductase; PUFA, polyunsaturated fatty acid; PUFA-PLs, polyunsaturated fatty acid-phospholipids; PUFA-PLs-OO^.^, PUFA-PLs peroxyl radicals; PUFA-PLs-OOH, PUFA-PLs peroxides; SODs, superoxide dismutases; TCA, tricarboxylic acid; TF, transferrin; TFRC, transferrin receptor.

Third, the tricarboxylic acid cycle and related metabolites in mitochondria facilitate ferroptosis by promoting the production of reactive oxygen species, ATP, or PUFA-PLs.^39^^,^^40^ Figure 1 illustrates the major pathways of the ferroptosis-inducing system.

### Ferroptosis defense system

Ferroptosis defense system can be divided into two categories: GPX4-dependent and GPX4-independent defense systems.

#### GPX4-dependent clearance of lipid peroxides

Glutathione peroxidases (GPXs) are a family of multiple isozymes that catalyze the reduction of H_2_O_2_ or organic hydroperoxides to water or corresponding alcohols using reduced glutathione (GSH) as an electron donor.^41^^,^^42^ GPX4 is the only GPX member capable of converting phospholipid hydroperoxides to phospholipid alcohols. Genetic deletion or pharmacological inhibition of GPX4 induces extensive accumulation of lipid peroxidation and triggers robust ferroptosis.^43^, ^44^, ^45^ GPX4 has three isoforms: cytosolic, mitochondrial, and nuclear GPX4, featured with different subcellular localizations.^46^^,^^47^ Among these three isoforms, cytosolic GPX4 is considered the major form in antagonizing ferroptosis,^48^ while recent studies suggest that mitochondrial GPX4 is also active in defending against ferroptosis in mitochondria by cooperating with dihydroorotate dehydrogenase (DHODH).^49^

GSH, the important substrate of GPX4, is a tripeptide consisting of glycine, glutamate, and cysteine.^50^^,^^51^ Cysteine is the rate-limiting amino acid for GSH synthesis, which is imported into cells in an oxidized dimeric form, cystine. Solute carrier family 7 member 11 (SLC7A11; also called xCT) is a core subunit of system x_c_^−^ that is a critical antiporter for the uptake of extracellular cystine in exchange for glutamate.^52^ Pharmacologically inhibition of SLC7A11-mediated cystine transport with erastin induces classic ferroptotic cell death in a GPX4-dependent manner.^20^ It is well accepted that the SLC7A11–GSH–GPX4 axis constitutes the major cellular defense system against ferroptosis. However, some cancer cell lines can still survive upon ferroptosis induction even in GPX4 deficient conditions, suggesting the presence of alternative ferroptosis defense machinery.^53^

#### GPX4-independent removal of lipid peroxides

Recent studies revealed that there are at least four additional signaling pathways that could suppress ferroptosis in the absence of GPX4: NADPH–ferroptosis suppressor protein 1 (FSP1)–reduced form of coenzyme Q10 (CoQH_2_), NADPH–FSP1–vitamin K hydroquinone, DHODH–CoQH_2_, and GTP cyclohydrolase 1 (GCH1)–tetrahydrobiopterin (BH4) pathways. FSP1 is a NAD(P)H-dependent oxidoreductase capable of reducing CoQ to CoQH_2_.^53^^,^^54^ CoQH_2_ has been proven to trap lipid peroxyl radicals to reduce lipid peroxidation and ferroptosis, which is independent of its function in mitochondrial electron transport.^53^, ^54^, ^55^ More recently, FSP1 was discovered to effectively convert vitamin K to its hydroquinone form, a powerful radical-capturing antioxidant and suppressor of (phospho)lipid peroxidation.^56^, ^57^, ^58^ DHODH is an enzyme involved in pyrimidine synthesis, which can reduce CoQ to CoQH_2_ in the inner mitochondrial membrane.^49^ Mitochondrial GPX4 and DHODH compensate for each other to control the level of mitochondrial lipid peroxidation.^49^ When GPX4 is inactivated, the DHODH activity and expression are dramatically increased, resulting in more production of CoQH_2_ to neutralize lipid peroxidation.^49^

BH4 is a radical-scavenging antioxidant that can trap lipid peroxyl radicals, and its ability to inhibit ferroptosis seems to be separate from its role as an enzymatic cofactor.^59^ GCH1 mediates the rate-limiting reaction in the biosynthesis pathway of BH4.^60^ It has been revealed that GCH1 protects cells against ferroptosis by producing BH4 as a radical-scavenging antioxidant as well as by generating CoQH_2_ through GCH1-mediated synthesis.^59^

**Figure 2:**
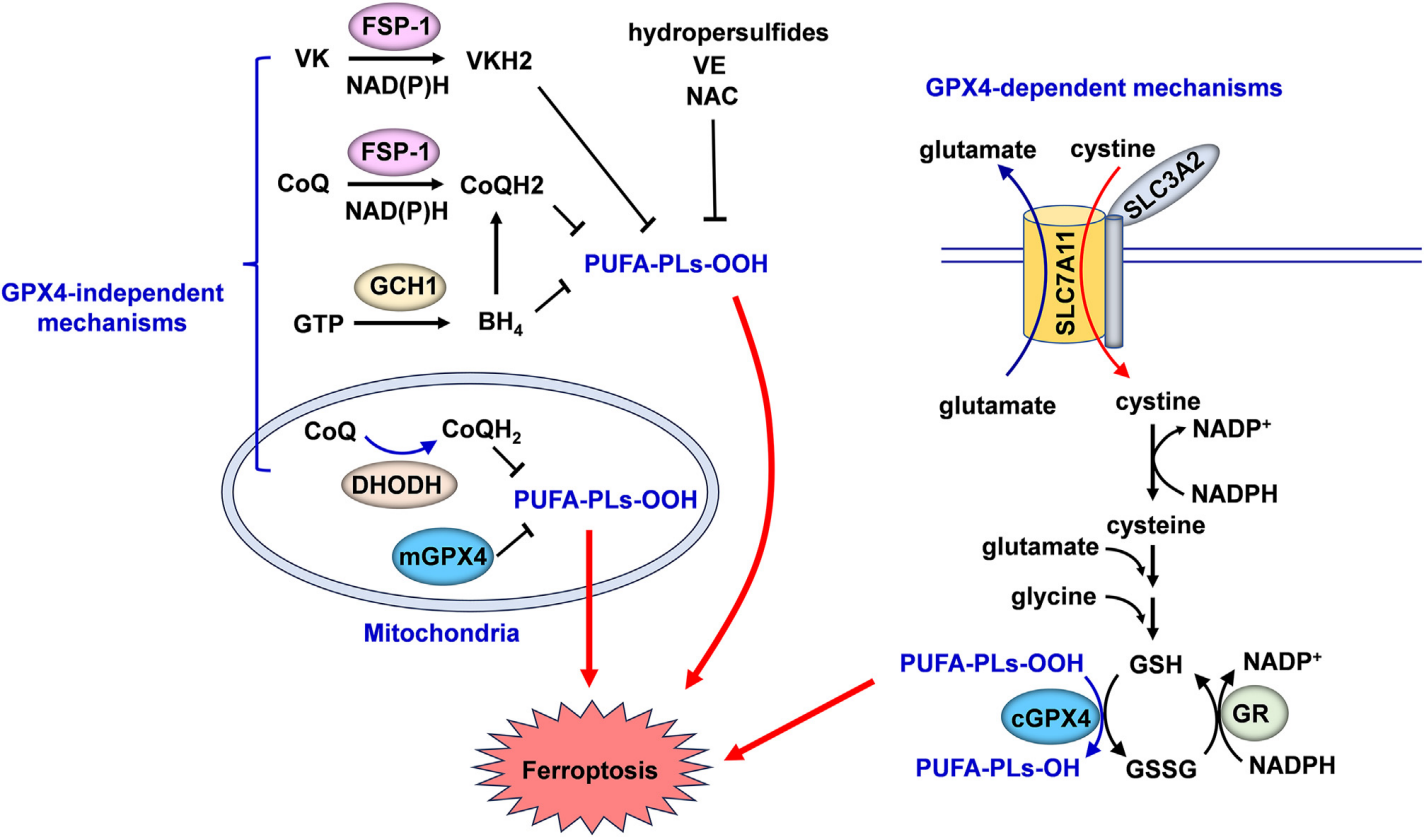
Ferroptosis defense system. Ferroptosis defense system can be divided into two categories: GPX4-dependent and GPX4-independent mechanisms. SLC7A11–GSH–GPX4 axis constitutes the major cellular defense system against ferroptosis. In addition, there are at least four additional signaling pathways that could suppress ferroptosis in the absence of GPX4: NADPH–FSP1– CoQH_2_, NADPH–FSP1–VKH_2_, DHODH–CoQH_2_, and GCH1–BH4 pathways. Moreover, vitamin E, n-acetyl cysteine, and hydropersulfides have been reported to serve as endogenous small molecular inhibitors of ferroptosis. BH4, tetrahydrobiopterin; CoQ, coenzyme Q10; CoQH_2_, reduced form of CoQ; cGPX4, cytosolic glutathione peroxidase 4; DHOOH, dihydroorotate dehydrogenase; FSP1, ferroptosis suppressor protein 1, also known as AIFM2; GCH-1, GTP cyclohydrolase 1; GR, glutathione reductase; GSH, glutathione; GSSG, oxidized glutathione; mGPX4, mitochondrial GPX4; NAC, N-acetylcysteine; VE, vitamin E; VK, vitamin K; VKH2, vitamin K hydroquinone (reduced form of VK);.

In addition to GSH, CoQH_2_, BH4, and vitamin K hydroquinone, vitamin E, n-acetyl cysteine, and hydropersulfides have been reported to serve as endogenous small molecular inhibitors of ferroptosis.^61^, ^62^, ^63^ Figure 2 illustrates the major pathways of the ferroptosis defense system.

## SLC7A11, stress, and ferroptosis

### Introduction of SLC7A11

System x_c_^−^ is a membrane-bound antiporter responsible for transporting cystine into cells in exchange for glutamate. It consists of the transmembrane transporter protein SLC7A11, which has 12 membrane-spanning segments, and the regulatory subunit SLC3A2, which has a single transmembrane domain and is connected to SLC7A11 via a disulfide bridge.^64^^,^^65^ SLC7A11 plays a critical role in cellular redox balance and antioxidant defense. The transported cystine by SLC7A11 is a critical component for the synthesis of glutathione, a potent antioxidant that protects cells from oxidative stress.^66^ Glutathione helps neutralize reactive oxygen species and detoxify harmful compounds. Of note, GPX4 utilizes glutathione as a co-factor in its enzymatic activity to eliminate lipid peroxides by converting reduced glutathione to its oxidized form in the process.^50^^,^^67^

The expression and activity of SLC7A11 can be regulated by various factors and stresses, including oxidative stress, nutrient availability, ER stress, radiation, oncogenic stress, DNA damage, and immune stress. Dysregulation of SLC7A11 has been implicated in various diseases, including cancer, neurodegenerative disorders, and metabolic disorders.^18^^,^^68^, ^69^, ^70^

### Structure of SLC7A11

**Figure 3:**
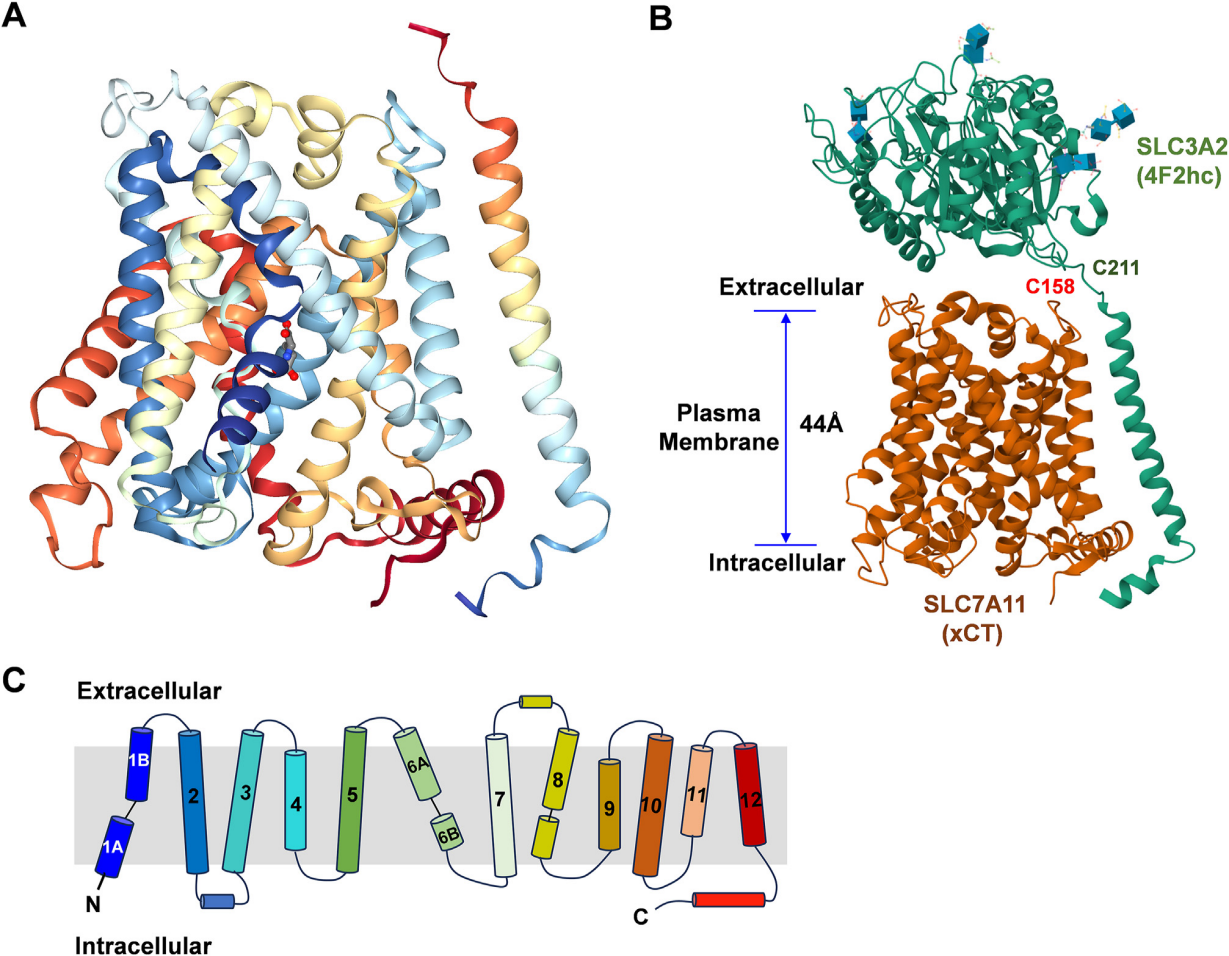
Cryogenic electron microscopy structure of SLC7A11. **(A)** Cartoon representation of SLC7A11 structure in complex with glutamate. SLC7A11 is colored blue from the N-terminus to red at the C-terminus. Glutamate is positioned at the core of the protein, marked in red. Protein Data Bank (PDB) ID: 7P9U. **(B)** Cartoon representation of system x_c_^−^ that consists of the transmembrane transporter protein SLC7A11 (dark red, with 12 membrane-spanning segments) and the regulatory subunit SLC3A2 (green). The cysteines (C158 and C211) form a disulphide bond to link the two subunits. PDB ID: 7P9V. **(C)** Diagram of transmembrane helixes (TM1 to TM12) of SLC7A11.

As shown in the high-resolution cryogenic electron microscopy 3D structure of SLC7A11 (Fig. 3A, B),^71^ the core subunit of the human system x_c_^−^ transporter, SLC7A11 (xCT), comprises 12 transmembrane helices adopting the canonical APC superfamily fold. The structure also reveals the transporter's regulatory subunit, SLC3A2 (4F2hc), which is connected to SLC7A11 through a disulphide bond between Cys211 in SLC3A2 and Cys158 in SLC7A11. In this latest study on SLC7A11 structure, by comparison of the cryogenic electron microscopy structure of system x_c_^−^ in both the apo and glutamate bound states, it reveals an allosteric mechanism for ligand discrimination: ligand binding induces an allosteric transition in transmembrane helix (TM) 8, which aids in glutamate recognition and the closure of the intracellular gate through the interaction of TM1A and TM6B with TM8. Upon switching to the outward open state, the transporter releases glutamate, and TM8 is speculated to unwind around Gly334, similar to the state observed in the inward open apo structure, facilitating l-cystine entry. Cystine then coordinates the movement of the mobile gate helices, TM1 and TM6, along with the hash domain. This involves the interaction of Arg135 on TM3 and Arg396 on TM10 with Tyr244 on TM6A to seal the cytoplasmic gate. TM1A moves away from the hash domain, opening the binding site to the cytoplasm for cystine to exit and be reduced to l-cysteine. In the high concentration of l-glutamate in the cytoplasm, l-glutamate rapidly enters the binding site, completing the antiport cycle.^71^

### Slc7a11 knockout mice

Sato et al generated the *slc7a11* knockout mice and observed that the *slc7a11*^*−/−*^ mice appeared healthy and were capable of reproduction, yet they exhibited elevated cystine levels in their plasma compared with *slc7a11*^*+/−*^ or *slc7a11*^*+/+*^ mice. Additionally, the plasma GSH levels in these mice were lower than in *slc7a11*^*+/+*^ mice. When cultured in standard media, embryonic fibroblasts from *slc7a11*^*−/−*^ mice did not survive; however, their survival and growth could be rescued by adding 2-mercaptoethanol or N-Acetyl cysteine.^64^ Their study suggests that while system x_c_^−^ is essential for maintaining plasma redox balance in living organisms, it is not crucial for mammalian development but is critical for cell viability and growth *in vitro*.^64^

The study of Wang et al further revealed that the absence of the *slc7a11* gene does not inherently induce ferroptosis under basal conditions.^72^ However, it does make these mice more susceptible to ferroptosis when there is an iron overload. This increased susceptibility is due to the impaired uptake of cystine and a consequent rise in reactive oxygen species production.^72^ Therefore, the *slc7a11* gene appears to protect ferroptosis in situations of iron overload.

### Regulation of SLC7A11 by various stressors

#### Nutrient starvation

Several studies have shown that nutrient deprivation can induce SLC7A11 expression. For example, Guang et al reported that glucose starvation induces SLC7A11 expression relying on transcription factors activating transcription factor 4 (ATF4) and NFE2 like bZIP transcription factor 2 (NRF2 or NFE2L2).^73^ In addition, deprivation of some amino acids (*e.g.*, cystine, arginine, tryptophan, histone, serine, and glycine) not only up-regulates the expression level of SLC7A11 but also enhances its antiporter activity.^74^^,^^75^

Recently, a group reported that pancreatic ductal adenocarcinoma cells employ the autophagy machinery to control the activity and localization of SLC7A11 on the cell's plasma membrane. Inhibition of autophagy re-localizes the SLC7A11 to lysosomes and inactivates it in an mTOR complex 2-dependent manner.^76^ Consistently, another group reported that mTOR complex 2, a sensor of growth factor signaling, interacts with and phosphorylates SLC7A11 at serine 26, resulting in a suppression of its transporter activity and consequent reduced GSH level and increased sensitivity to ferroptosis.^77^

It is widely accepted that SLC7A11 up-regulation increases cysteine intake and prevents ferroptosis, which benefits cell survival in a detrimental condition. However, recently, Gan's group proposed a new perspective that cancer cells expressing high levels of SLC7A11 may be unfavorable for their survival in a glucose starvation or glutamine starvation condition.^78^^,^^79^ The logic is as below: high expression of SLC7A11 will constitutively import cystine into cells, which has to be reduced to a more soluble and nontoxic form, cysteine. This process continuously consumes and depletes the cellular NADPH that is mainly synthesized by the pentose phosphate pathway of glucose metabolism. Upon glucose starvation, the NADPH pool is dramatically decreased, leading to aberrant accumulation of intracellular cystine and a new form of cell death disulfidptosis.^78^^,^^80^

#### Oxidative stress

BTB and CNC homology 1 (BACH1) and NRF2 are important oxidative stress-responsive proteins.^81^^,^^82^ They are transcription factors that regulate a series of common antioxidative genes such as SLC7A11 and heme oxygenase-1.^83^^,^^84^ It is well established that BACH1 competes with NRF2 for binding to the antioxidant response elements. As a result, BACH1 inhibits the expression of anti-oxidative genes, which are activated by NRF2 during oxidative stress responses.^82^

Oxidative stress stabilizes and activates NRF2 in the cell nucleus, and the hyperactivation of NRF2 induced SLC7A11 expression by directly binding to its promoter, leading to reduced ferroptosis and enhanced radioresistance.^85^^,^^86^ In contrast, oxidative stress inhibits the DNA binding activity of BACH1 and promotes BACH1 nuclear export and degradation, thereby inducing SLC7A11 and other anti-oxidative gene expression.^87^ Consistently, antioxidants (*e.g.*, N-acetylcysteine and vitamin E) can decrease free heme levels and stabilize BACH1.^88^ NRF2–SLC7A11 and BACH1–SLC7A11 signaling pathways have been demonstrated to serve important but opposite roles in regulating ferroptosis.^12^^,^^83^^,^^89^ In contrast, oxidative stress induced by erastin has been found to induce activating transcription factor 3 (ATF3) to directly suppress SLC7A11 transcription.^90^

#### Radiation stress

Recently, radiation has been recognized to induce ferroptosis.^13^^,^^91^ Interesting, radiation also rapidly induce SLC7A11 expression in a time-dependent manner, which could be an adaptive response to antagonize radiation-induced ferroptotic cell death and tissue damage.^13^ While the specific process that leads to the increased expression of SLC7A11 following radiation is not yet clear, it probably involves the activation of NRF2 and ATF4. Both NRF2 and ATF4 are typically activated by radiation and are recognized as regulators of SLC7A11 transcription.^92^^,^^93^ Conversely, radiation-induced p53 activation down-regulates radiation-induced SLC7A11 expression and sensitizes cells to radiation-induced ferroptosis. It is proposed that p53 deficiency-mediated radioresistance in cancer cells is at least partially through SLC7A11-mediated ferroptosis suppression.^94^ In addition, ATF3 is also induced upon radiation to directly suppress SLC7A11 transcription.^90^^,^^95^

#### DNA damage responses

DNA damage refers to any alteration or modification that occurs in the structure or sequence of DNA molecules.^96^ The causes for DNA damage include but are not limited to radiation, chemicals, replication errors, and oxidative stress.^96^ p53 protein level is rapidly increased in response to DNA damage through a post-translational regulation mechanism.^97^ Jiang et al revealed that p53 inhibits cystine uptake and sensitizes cells to ferroptosis by repressing the transcription of SLC7A11.^98^ In addition to affecting the uptake of cystine, Chu et al further found that SLC7A11 specifically interacts with 12-lipoxygenase and inhibits its enzymatic activity, which provides a distinct mechanism for p53-mediated sensitization of ferroptosis.^26^ Furthermore, DNA damage-mediated down-regulation of SLC7A11 may also result from the induction of ATF3.^95^ Xie et al reported that in a specific cell type, under certain drug treatment, p53 may exert a distinct role in the regulation of SLC7A11. This suggests that p53, as a central stress responder, may form different protein complexes in varied circumstances to regulate redox homeostasis.^99^

#### Oncogenic stress

Several important studies have reported that oncogenic stress can up-regulate SLC7A11 level to control the redox balance and repress ferroptosis, which could be favorable for tumor transformation and progression. For example, Hu et al reported that mutant KRAS proto-oncogene, GTPase (KRAS) increases cellular cystine levels and glutathione biosynthesis.^100^ It is shown that SLC7A11 is overexpressed in patients with KRAS-mutant lung adenocarcinoma and has a positive correlation with tumor progression.^100^ A similar study by Lim et al showed that constitutive induction of the RAS-RAF-MEK-ERK signaling cascade directly transactivates the SLC7A11 promoter by downstream activation of ETS proto-oncogene 1, transcription factor (ETS1) in synergy with another transcriptional factor ATF4.^101^

Interestingly, NRF2 is not only an oxidative stress responder, but also an oncogenic stress responder, which can be transcriptionally up-regulated by oncoproteins Myc, KRAS, and BRAF.^102^^,^^103^ Elevated NRF2 level subsequently enhances the expression of SLC7A11 and other endogenous antioxidants.^89^^,^^102^

Recently, our group found that hotspot p53 mutant p53^R175H^ up-regulates SLC7A11 through a gain-of-function mechanism. Specifically, p53^R175H^ cannot directly activate SLC7A11 promoter, but it up-regulates SLC7A11 expression by suppressing BACH1-mediated repression of SLC7A11 transcription. Moreover, we discovered that p53^R175H^-induced up-regulation of SLC7A11 enhances the resistance of tumor cells to ferroptosis *in vivo*, which at least partially contributes to p53^R175H^-mediated tumor growth.^12^

#### ER stress

Although ATF4 is also up-regulated by nutrient deprivation, the main inducing factor is endoplasmic reticulum (ER) stress.^73^ ER stress induces ATF4 expression, which in turn transcriptionally activates SLC7A11 to suppress ferroptosis and the resulting inflammation. The ATF4–SLC7A11 axis has been proven to be important for the prevention of liver damage and hepatocarcinogenesis.^104^ In contrast, erastin-induced ER stress contributes to ATF3 induction and subsequently represses the transcription of SLC7A11.^90^

#### Immune stress

Wang et al reported that activated CD8^+^ T cells in immunotherapy enhance lipid peroxidation in tumor cells, leading to increased ferroptotic cell death which contributes to the effectiveness of immunotherapy. This process involves the release of interferon gamma (IFNγ) from CD8^+^ T cells, which down-regulates the expression of SLC3A2 and SLC7A11, components of the glutamate-cystine antiporter system x_c_^−^, thereby inhibiting cystine uptake by tumor cells and promoting lipid peroxidation and ferroptosis in those cells.^14^ Further study showed that IFNγ-induced activation of Janus kinase 1/2 (JAK1/2)–signal transducer and activator of transcription 1 (STAT1) pathway could be critical for the repression of SLC7A11 expression.^14^^,^^105^

**Figure 4:**
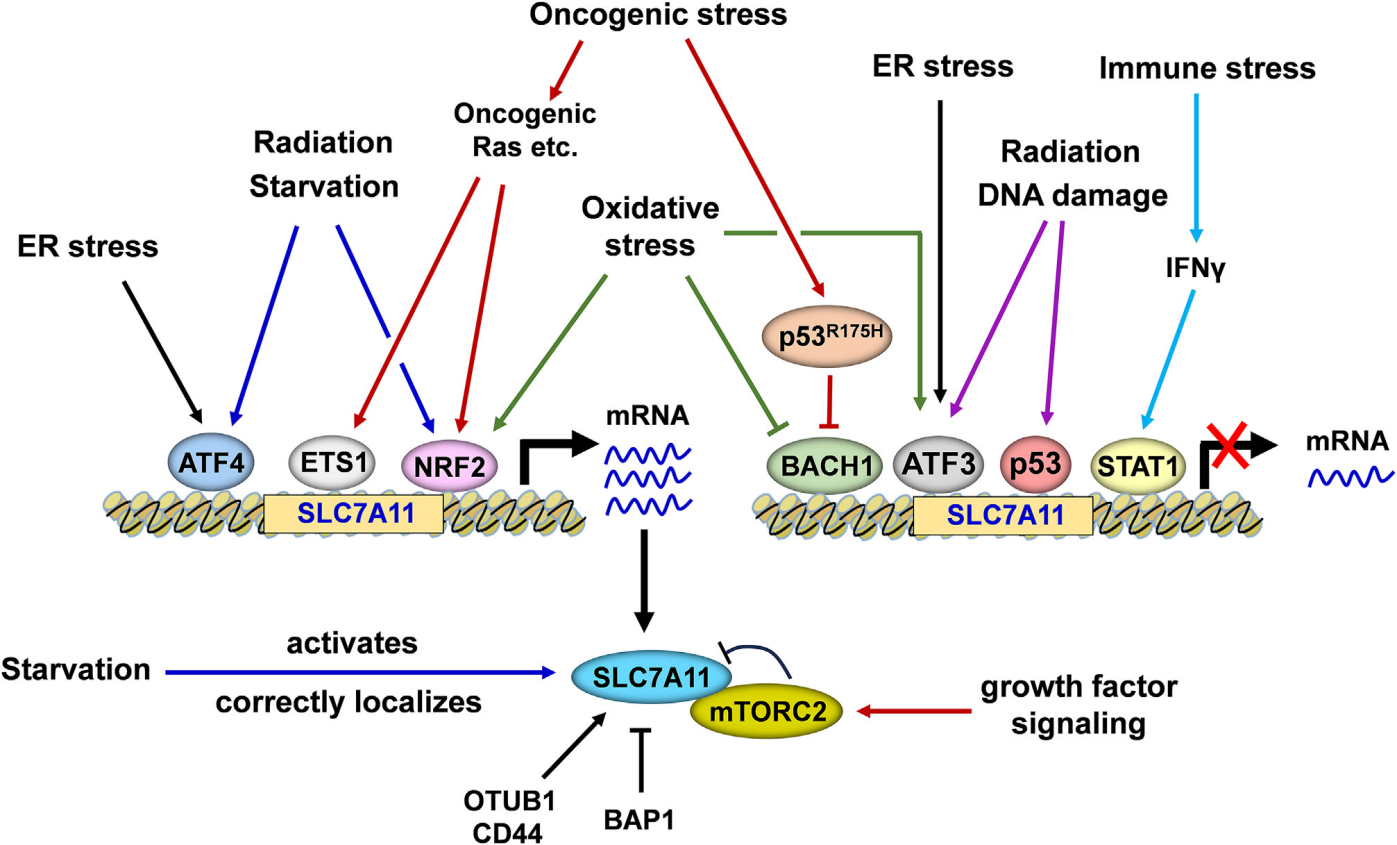
Regulation of SLC7A11 by distinct stresses. ATF4, ETS1, and NRF2 act as transcriptional activators of SLC7A11, and their expression or activity is enhanced in response to ER stress, radiation, starvation, oncogenic stress, and oxidative stress. In contrast, BACH1, p53, ATF3, and STAT1 serve as transcriptional suppressors of SLC7A11, with their level or activity being regulated by radiation, DNA damage, immune stress, oxidative stress, and oncogenic stress. Additionally, certain stresses or factors (OTUB1, CD44, and BAP1^17^^,^^124^) are involved in the post-translational regulation of SLC7A11. ATF3, activating transcription factor 3; ATF4, activating transcription factor 4; BACH1, BTB domain and CNC homolog 1; ETS1, ETS proto-oncogene 1, transcription factor; IFN γ, interferon γ; mTORC2, mTOR complex 2; mTORC2, mechanistic target of rapamycin complex 2; NRF2, also called NFE2L2, NFE2 like bZIP transcription factor 2; OTUB1, OTU deubiquitinase, ubiquitin aldehyde binding 1; STAT1, signal transducer and activator of transcription 1.

In summary, SLC7A11 is a sensitive responder to various stresses. Among these stresses, nutrient starvation and oncogenic stress tend to induce SLC7A11 expression or activate its transporter activity, while DNA damage and immune stress appear to suppress SLC7A11 expression. Other stresses such as oxidative stress, radiation, and ER stress show dual functions in the regulation of SLC7A11 through distinct transcription factors. Therefore, whether a stress up-regulates or down-regulates SLC7A11 is dependent on the specific type of the stress and the microenvironment. The major regulation of stresses and SLC7A11 are shown in Figure 4.

## Targeting SLC7A11 for cancer therapy

As discussed above, SLC7A11 is a sensitive and vital responder to multiple stresses and serves as an unconventional checkpoint in tumorigenesis by modulating ferroptotic responses. Up-regulation or activation of SLC7A11 can increase the fitness of cancer cells, leading to the lasting progression of a tumor in an unfavorable environment. This leads to a prevalent therapeutic concept: targeting SLC7A11 for cancer treatment.

### SLC7A11 expression in normal tissues versus tumor tissues

**Figure 5:**
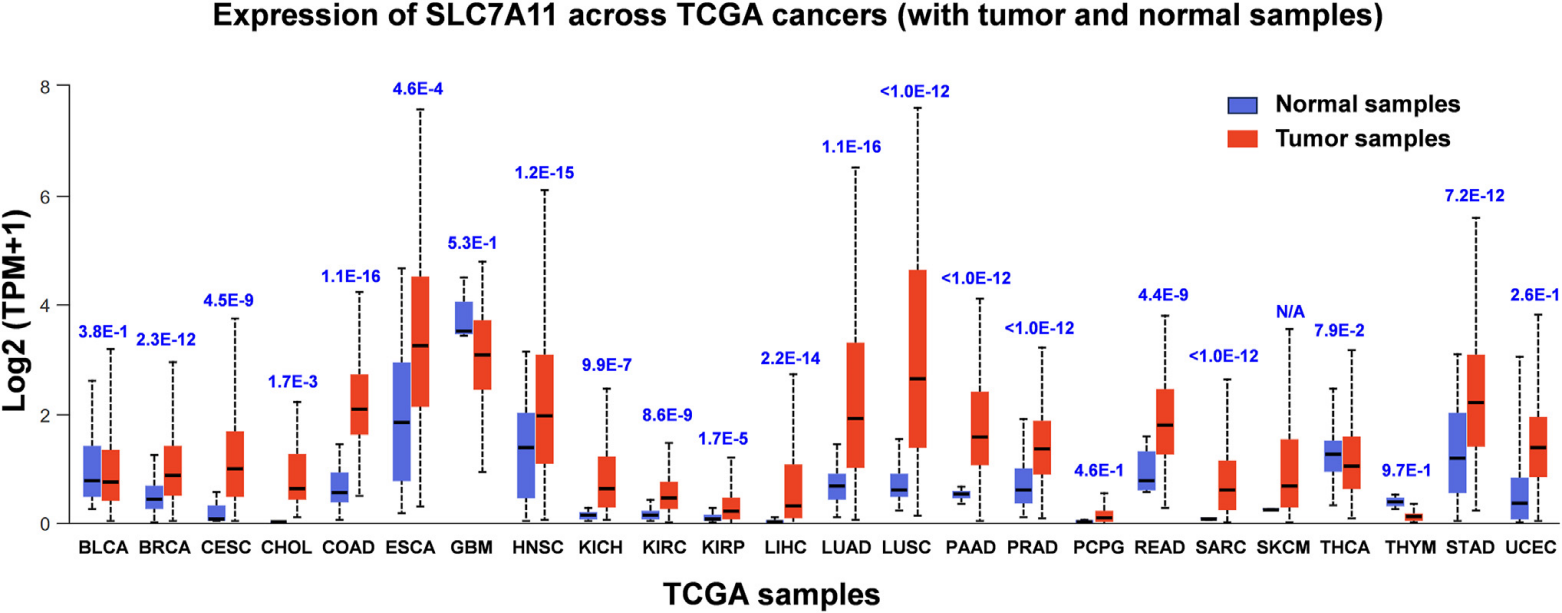
Expression of SLC7A11 in normal tissues versus tumor tissues. Patients' expression data are from the TCGA database and analyzed using UALCAN (https://ualcan.path.uab.edu). *P* values are displayed at the top of each cancer type. BLCA, bladder urothelial carcinoma; BRCA, breast invasive carcinoma; CESC, cervical squamous cell carcinoma and endocervical adenocarcinoma; CHOL, cholangiocarcinoma; COAD, colon adenocarcinoma; ESCA, esophageal carcinoma; GBM, glioblastoma multiforme; HNSC, head and neck squamous cell carcinoma; KICH, kidney chromophobe; KIRC, kidney renal clear cell carcinoma; KIRP, kidney renal papillary cell carcinoma; LIHC, liver hepatocellular carcinoma; LUAD, lung adenocarcinoma; LUSC, lung squamous cell carcinoma; PAAD, pancreatic adenocarcinoma; PRAD, prostate adenocarcinoma; PCPG, pheochromocytoma and paraganglioma; READ, rectum adenocarcinoma; SARC, sarcoma; SKCM, skin cutaneous melanoma; STAD, stomach adenocarcinoma; THCA, thyroid carcinoma; THYM, thymoma; UCEC, uterine corpus endometrial carcinoma.

By analyzing the TCGA patient database, it has been found that SLC7A11 mRNA expression is significantly higher in tumor tissues than in normal tissues across most cancer types. This includes high-incidence cancers such as breast invasive carcinoma, colon adenocarcinoma, liver hepatocellular carcinoma, lung adenocarcinoma, squamous cell carcinoma, pancreatic adenocarcinoma, and prostate adenocarcinoma (Fig. 5).

### SLC7A11 expression and overall survival

**Figure 6:**
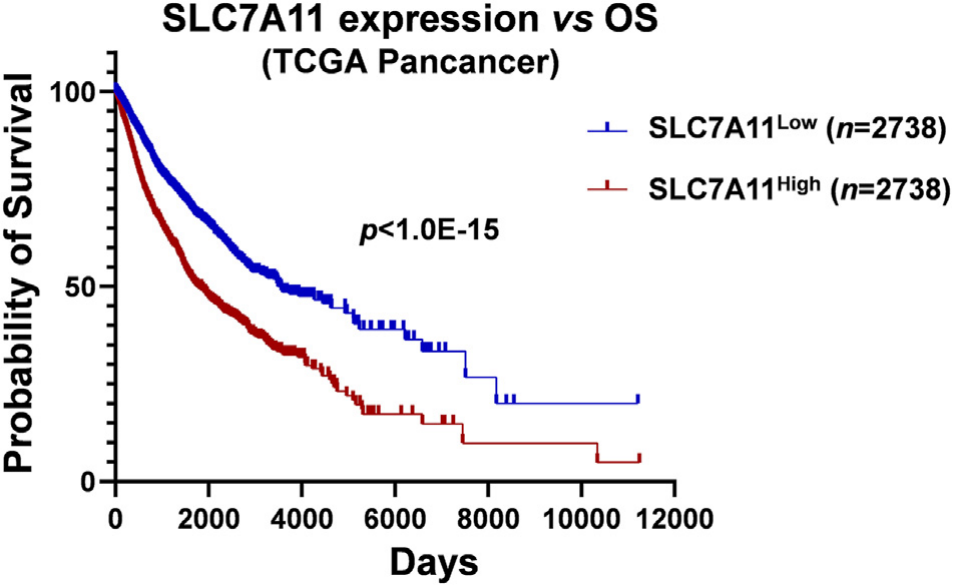
SLC7A11 expression and overall survival. Patients' SLC7A11 expression and survival data were obtained from the TCGA pan-cancer database (https://xenabrowser.net/). *P* values for the survival curves were determined using the Log-rank (Mantel–Cox) test, and the sample sizes (*n*, numbers) are displayed on the panel. Cutoff line for SLC7A11 expression is top 25% for high expression and last 25% for low expression.

Further analysis reveals a negative correlation between SLC7A11 expression and patients' overall survival. Pancancer data from the TCGA database clearly demonstrate that higher SLC7A11 expression in tumor tissues is associated with shorter overall survival of cancer patients (*P* < 1.0E-15) (Fig. 6). Consistently, survival data for specific cancer types, including breast invasive carcinoma, liver hepatocellular carcinoma, lung adenocarcinoma, and kidney cancer, also indicate that a higher SLC7A11 expression level predicts a worse prognosis (UALCAN, https://ualcan.path.uab.edu).

### Summary of SLC7A11 targeted cancer therapies

SLC7A11 has been identified as playing a significant role in various aspects of tumor progression, including tumorigenesis, proliferation, metastasis, prognosis, and chemoresistance.^18^^,^^65^^,^^98^^,^^106^ While recent research suggests that excessively high levels of SLC7A11 may increase the vulnerability of cancer cells to glucose starvation due to rapid NADPH consumption,^78^ the majority of pre-clinical studies indicate that elevated SLC7A11 expression in tumor cells enhances their adaptability to harsh environments, thereby facilitating tumor survival and progression. Increasing evidence supports the notion that targeting the SLC7A11 pathway with compounds or siRNA/miRNA presents a promising therapeutic approach for a wide range of tumors.

One important category of SLC7A11 targeted drugs is to directly suppress the amino acids transportation activity of SLC7A11 to reduce the intracellular GSH level. These drugs/compounds include sulfasalazine, erastin, imidazole ketone erastin, and HG106.^100^^,^^107^, ^108^, ^109^ For example, SLC7A11-mediated metabolic reprogramming promotes non-small cell lung cancer progression and inhibition of SLC7A11 transporter activity in SLC7A11 overexpressing non-small cell lung cancer cells with sulfasalazine decreases cell proliferation and invasion *in vitro* and *in vivo*.^107^ In addition, as the SLC7A11 is expressed on one-third of triple-negative breast tumors *in vivo*, sulfasalazine was proven to suppress tumor growth of triple-negative tumors.^106^ Erastin, a classic SLC7A11 inhibitor, was observed to induce intense cytotoxicity on colorectal cancer stem cells *in vitro* and *in vivo* and mitigated the chemoresistance of colorectal cancer stem cells.^108^ Imidazole ketone erastin is a pharmaceutically improved analogue of erastin with enhanced metabolic stability and solubility, which effectively suppresses the tumor growth in a diffuse large B cell lymphoma xenograft model. A nanoparticles-formulated imidazole ketone erastin has a similar therapeutic effect with imidazole ketone erastin alone but has lower toxicity *in vivo*.^109^ In addition, another novel SLC7A11 inhibitor, HG106, exhibited specific cytotoxicity against KRAS-mutant lung adenocarcinoma cells by inducing cell apoptosis through enhanced oxidative stress and ER stress pathways.^100^ Importantly, administration of HG106 to various preclinical mouse models of KRAS-mutant lung adenocarcinoma resulted in significant tumor suppression and prolonged survival.^100^

The other category of SLC7A11 targeted drugs is to reduce the mRNA or protein level of SLC7A11 directly or indirectly. For examples, miRNA-27a negatively regulates SLC7A11 expression in cisplatin-resistant bladder cancer.^110^ Similarly, administration of a nanoparticle-based siRNA targeting SLC7A11 effectively suppressed tumor growth, reduced metastasis occurrence, inhibited activation of cancer-associated fibroblasts, and mitigated fibrosis in orthotopic pancreatic ductal adenocarcinoma tumors.^111^ Shen et al reported that elevated expression of phosphoglycerol dehydrogenase (PHGDH) was observed in breast cancer patients. Silencing of PHGDH resulted in decreased expression of SLC7A11. Mechanistically, PHGDH interacts with PCBP2, an RNA-binding protein, impeding its ubiquitination and degradation. Consequently, PCBP2 stabilizes SLC7A11 mRNA, leading to increased expression. The PHGDH inhibitor NCT-502 effectively hinders tumor advancement in breast cancer by facilitating ferroptosis.^112^ Sculponeatin A, a diterpenoid extracted from *I. sculponeatus*, was observed to hamper the transcription of SLC7A11 by directly binding to and inhibiting the ETS1 protein, a well-known activator of SLC7A11 transcription. Furthermore, sculponeatin A facilitates the proteasomal degradation of ETS1. Notably, in a mouse model, sculponeatin A effectively suppresses tumor growth through ferroptosis without inducing apparent toxicity.^113^ Interestingly, vitamin D or 2-imino-6-methoxy-2H-chromene-3-carbothioamide was observed to down-regulate SLC7A11 expression to inhibit colorectal cancer growth through induction of ferroptosis.^114^^,^^115^ In addition, sulforaphane was reported to induce ferroptosis in small-cell lung cancer cells by inhibiting SLC7A11 expression, and sodium butyrate induces ferroptosis in endometrial cancer cells by decreasing the expression of SLC7A11 by enhancing the expression of a RNA binding protein RBM3.^116^^,^^117^

Moreover, different SLC7A11 inhibitors may synergistically suppress tumor progression. For example, metformin alone has been reported to decrease SLC7A11 protein stability by inhibiting its UFMylation, and metformin combined with sulfasalazine can cooperatively induce ferroptosis and suppress tumor growth in the breast cancer model.^118^ Hong et al reported that PARP inhibition by olaparib down-regulates SLC7A11 expression in a p53-dependent manner to trigger ferroptosis, and a combination of sulfasalazine and olaparib synergistically suppresses BRCA-proficient ovarian cancer cell growth and prolongs the mice's survival in a xenograft model.^119^ Moreover, inhibition of SLC7A11 by erastin, sulfasalazine, or nortriptyline hydrochloride (an inhibitor of SLC7A11 translation) has been found to improve the therapeutic effect of radiotherapy.^13^^,^^120^ The major studies on SLC7A11 targeted cancer therapy are listed in Table 1.

**Table 1:** A summary of recent progress in targeting SLC7A11 for cancer therapy.

Drugs/compounds	Targets	Mechanisms	Involves ferroptosis?	Cancer type	Effects	*In vitro*/*in vivo*	Reference
Sulfasalazine	SLC7A11	Inhibits SLC7A11 transporter activity	Not mentioned	Non-small cell lung cancer, triple-negative breast cancer	Decreases tumor proliferation and invasion	*In vivo*	106,107
Erastin	SLC7A11	Suppresses SLC7A11 transporter activity	Yes	Colorectal cancer	Attenuates the chemoresistance of colorectal cancer stem cells	*In vitro*	108
Imidazole ketone erastin	SLC7A11	Inhibits SLC7A11 transporter activity	Yes	Diffuse large B cell lymphoma	Imidazole ketone erastin or its nanoparticle drug inhibits xenograft tumor growth of diffuse large B cell lymphoma	*In vivo*	109
HG106; sulfasalazine	SLC7A11	Inhibits SLC7A11 transporter activity	Not mentioned	Lung adenocarcinoma	Induces tumor suppression in KRAS-mutant lung adenocarcinoma and prolongs survival	*In vivo*	100
miRNA-27a or sulfasalazine	SLC7A11	Targets SLC7A11	Not mentioned	Bladder cancer	Conquers cisplatin resistance	*In vitro*	110
SLC7A11 siRNA nanoparticle drug	SLC7A11	Reduces SLC7A11 expression	Not mentioned	Pancreatic ductal adenocarcinoma	Reduces tumor growth, incidence of metastases, and cancer-associated fibroblasts activation	*In vivo*	111
Sulforaphane	SLC7A11	Inhibits mRNA and protein expression levels of SLC7A11	Yes	Small-cell lung cancer	Inhibits cell growth and promote cell death in small-cell lung cancer	*In vitro*	116
NCT-502 (PHGDH inhibitor)	PHGDH- SLC7A11 pathway	Down-regulates SLC7A11 expression via affecting RNA-binding protein PCBP2	Yes	Bladder cancer	Inhibits tumor growth	*In vivo*	112
IMCA	SLC7A11	Down-regulates SLC7A11 expression	Yes	Colorectal cancer	Inhibits tumor growth	*In vivo*	115
Vitamin D	SLC7A11	Down-regulates SLC7A11 expression	Yes	Colorectal cancer	Promotes ferroptosis of colorectal cancer stem cells and suppresses tumor growth	*In vivo*	114
Sculponeatin A	ETS1-SLC7A11 pathway	Directly binds ETS1 to inhibit its transcriptional activity on SLC7A11 and promotes proteasomal degradation of ETS1	Yes	Breast cancer	Inhibits tumor growth without causing obvious toxicity	*In vivo*	113
Sodium butyrate	RBM3–SLC7A11 pathway	Indirectly down-regulates SLC7A11 expression by promoting the RBM3 expression	Yes	Endometrial cancer	Inhibits the tumor growth of endometrial cancer	*In vivo*	117
Metformin alone or combined with sulfasalazine	UFMylation of SLC7A11	Reduces SLC7A11 protein stability by inhibiting UFMylation	Yes	Breast cancer	Sulfasalazine synergistically enhances the anti-tumor activity of metformin	*In vivo*	118
Olaparib (PARP inhibitor) alone or combined with sulfasalazine	PARP-p53-SLC7A11 pathway	Down-regulates SLC7A11 expression in a p53-dependent manner	Yes	BRCA wild-type ovarian cancer	Inhibits tumor growth and increases survival	*In vivo*	119
miR-128-3p mimics + erastin	miR-128-3p/SLC7A11 pathway	miR-128-3p inhibits SLC7A11 expression	Yes	Prostate cancer	miR-128-3p decreases cell viability by enhancing ferroptosis	*In vitro*	122
NTP alone or combined with erastin	RBMS1-SLC7A11 pathway	RBMS1 ablation inhibits the translation of SLC7A11	Yes	Lung cancer	NTP sensitizes radioresistant lung cancer cells to radiotherapy	*In vitro*	120
Ursolic acid combined with sorafenib	SLC7A11 MCL1	Reduces SLC7A11 protein level and degrades MCL1	Yes	Pan-cancer	Inhibits tumor growth	*In vivo*	123
Ionizing radiation + erastin; ionizing radiation + sulfasalazine	SLC7A11	Erastin or sulfasalazine inhibits ionizing radiation-induced SLC7A11 activation	Yes	Pan-cancer	Sensitizes radioresistant cancer cells and xenograft tumors to ionizing radiation	*In vivo*	13

Notes: IMCA, 2-imino-6-methoxy-2H-chromene-3-carbothioamide; MCL1, MCL1 apoptosis regulator, BCL2 family member; NTP, nortriptyline hydrochloride; RBM3, RNA binding motif protein 3; RBMS1, RNA binding motif single-stranded interacting protein 1.

## Conclusion

In summary, this review highlights the pivotal role of SLC7A11 as a non-conventional checkpoint in the pathogenesis of cancer, primarily through its regulation of ferroptotic responses under a spectrum of stress conditions. SLC7A11, functioning as a transmembrane cystine/glutamate transporter, is delicately involved in the balance of cell survival and death, with its expression and function being finely tuned by various stressors including oxidative, nutrient, ER, radiation, oncogenic, DNA damage, and immune stresses. The dynamic regulation of SLC7A11 is through a complex interplay of transcriptional activators and repressors, alongside modulations in its activity, protein stability, and cellular localization. Notably, the overexpression of SLC7A11 in a range of human cancers correlates with poorer prognosis, further indicating its importance in oncogenic processes. The therapeutic potential of targeting SLC7A11 is immense, offering promising strategies for inhibiting tumor progression at multiple stages. This review emphasizes the critical role of SLC7A11 in the intricate network of metabolic regulation within the landscape of tumorigenesis.

## Future directions

In this review, we have presented that SLC7A11 acts as a central node connecting ferroptosis with various types of stress. However, it remains unknown why certain stresses up-regulate SLC7A11 expression, while others down-regulate it, and still others show a dual function in the regulation of SLC7A11. What is the physiological relevance of these differential regulations, and how does cross-talk occur in complex conditions involving multiple stresses?

SLC7A11's transcription is activated by ATF4, NRF2, and ETS1, but suppressed by BACH1, p53, ATF3, and STAT1 in reaction to diverse stresses. How do these factors collaborate or compete with each other at the SLC7A11 promoter?

SLC7A11 promotes the synthesis of glutathione to neutralize cellular reactive oxygen species and protect cells from ferroptotic cell death. However, a recent study reported that a high level of SLC7A11 may deplete the redox system of cancer cells, especially in glucose-starvation conditions, which could be detrimental to cancer cells.^78^ This raises a question: how do cancer cells balance these two contradictory effects? Moreover, it would be interesting to determine the exact conditions under which SLC7A11-based targeted therapy is applicable.

Inhibition of SLC7A11 facilitates ferroptosis in most cases but promotes apoptosis in certain cases.^100^^,^^109^ What are the mechanisms underlying the differential regulation of ferroptosis versus apoptosis by SLC7A11?

Current SLC7A11 inhibitors still have some limitations in specificity, efficiency, and half-life. Therefore, it would be meaningful to develop more specific and potent SLC7A11 inhibitors or degrading drugs that are applicable for *in vivo* use. As the 3D structure of SLC7A11 has been clarified, computer-aided drug design may be considered to enhance the specificity. Additionally, exploration of the combined use of SLC7A11 inhibitors with existing chemotherapy agents, targeted therapy drugs, and immune checkpoint inhibitors may offer enhanced anti-tumor efficacy.

Furthermore, it is worth noting that GSH can function as an endogenous copper chelator,^121^ adding an intriguing dimension to the investigation of SLC7A11's role in cellular homeostasis. Given the established role of SLC7A11 in redox regulation and its involvement in maintaining cellular antioxidant defenses, it raises the question of whether SLC7A11 may also influence cellular responses to copper-induced stress. The potential interplay between SLC7A11 and copper metabolism presents an exciting avenue for further research. Exploring this connection may provide valuable insights into the mechanisms behind copper-induced cell death and broaden our understanding of the multifaceted roles SLC7A11 plays in safeguarding cellular health.

## Author contributions

Z.S. and W.G. conceived the topic, discussed its contents, and wrote the manuscript. Y.L. provided some analyses of data. L.W. illustrated part of the figures.

## Conflict of interests

The authors declare that they have no competing financial interests.

## Funding

This work was supported by the National Cancer Institute of the National Institutes of Health (USA) (No. R35CA253059, RO1CA258390, RO1CA254970 to W.G.).
